# Infectivity of exhaled SARS-CoV-2 aerosols is sufficient to transmit covid-19 within minutes

**DOI:** 10.1038/s41598-023-47829-8

**Published:** 2023-12-01

**Authors:** Malin Alsved, Kristina Nyström, Sara Thuresson, David Nygren, Marianela Patzi-Churqui, Tareq Hussein, Carl-Johan Fraenkel, Patrik Medstrand, Jakob Löndahl

**Affiliations:** 1 Division of Ergonomics and Aerosol Technology, Department of Design Sciences, Lund University, Box 118, 22100 Lund, Sweden; 2 Department of Clinical Microbiology, Sahlgrenska University Hospital, Region Västra Götaland, 41346 Gothenburg, Sweden; 3 Department of Infectious Diseases, Institute of Biomedicine, Sahlgrenska Academy, University of Gothenburg, 41346 Gothenburg, Sweden; 4 Division of Infection Medicine, Department of Clinical Sciences, Lund University, 22100 Lund, Sweden; 5 Environmental and Atmospheric Research Laboratory (EARL), Department of Physics, School of Science, The University of Jordan, Amman, 11942 Jordan; 6 Institute for Atmospheric and Earth System Research (INAR/Physics), University of Helsinki, 00014 Helsinki, Finland; 7 Department of Clinical Microbiology and Infection Control, Region Skåne, 22185 Lund, Sweden; 8 Department of Translational Medicine, Clinical Virology, Lund University, 22100 Lund, Sweden; 9 SciLifeLab, Lund University, 22100 Lund, Sweden

**Keywords:** Translational research, SARS-CoV-2, Viral transmission

## Abstract

Exhaled SARS-CoV-2-containing aerosols contributed significantly to the rapid and vast spread of covid-19. However, quantitative experimental data on the infectivity of such aerosols is missing. Here, we quantified emission rates of infectious viruses in exhaled aerosol from individuals within their first days after symptom onset from covid-19. Six aerosol samples from three individuals were culturable, of which five were successfully quantified using TCID_50_. The source strength of the three individuals was highest during singing, when they exhaled 4, 36, or 127 TCID_50_/s, respectively. Calculations with an indoor air transmission model showed that if an infected individual with this emission rate entered a room, a susceptible person would inhale an infectious dose within 6 to 37 min in a room with normal ventilation. Thus, our data show that exhaled aerosols from a single person can transmit covid-19 to others within minutes at normal indoor conditions.

## Introduction

The transmission routes that enabled the efficient spread of covid-19 have been debated, but it has become evident that short-range aerosol transmission has contributed significantly^1,2^. However, as the infectivity peaks at or even before symptom onset it has been challenging to collect experimental data on the quantified infectivity of exhaled SARS-CoV-2 aerosols.

Three previous studies have reported attempts to cultivate exhaled aerosol samples from patients with covid-19. In one of these, no aerosol samples were positive when cultured^3^, but the other two studies reported qualitative results of culture-positive virus in exhaled air^4,5^. Two additional studies successfully quantified SARS-CoV-2 infectivity of aerosol samples^6,7^. However, these were from room or car air, and collected over hours which made it difficult to derive emission rates. Moreover, Kitagawa et al. recently measured the 50% tissue culture infectious dose (TCID_50_) in air samples from a hospital patient room, but did not calculate individual emission rates^8^. To our knowledge, no quantification has been done on virus isolated from exhaled aerosols of infected individuals. Nevertheless, this data is crucial for exposure assessments. Thus, critical information on emissions of infectious SARS-CoV-2 from exhaled air is still missing.

Source emission rates are crucial for modelling airborne transmission, which is key to estimate the risk for infection in different settings. Previously, we described an indoor air model for calculating the inhaled dose rate for SARS-CoV-2^9^. However, calculation of exposure time to acquire an infection was uncertain as information was missing about infectious dose, size of the virus-containing aerosol particles and emission rates of SARS-CoV-2. These missing pieces of information are now available. Recently, a human challenge study instilled virus in the nose of human test subjects and presented a quantitative value of TCID_50_ representing one infectious dose, *ID*_*50*_, for SARS-CoV-2^10^. There is also new information available on the particle size of virus containing aerosols^11^. Thus, the quantified infectivity of exhaled SARS-CoV-2 is the remaining key to estimating the exposure time needed to acquire infection for people in contact with someone who exhales infectious SARS-CoV-2.

The aim of this study was to measure the emission rate of infectious exhaled SARS-CoV-2 and thereafter estimate the time needed to inhale an infectious dose. We isolated SARS-CoV-2 from exhaled aerosol samples collected in a previous study^12^ and quantified their infectivity. Finally, we calculated the inhaled deposited dose based on a previously described indoor air transmission model^9^.

## Results

We quantified the infectivity of exhaled aerosol samples that were collected in a previous study^12^ during 10 min of breathing, talking or singing. Infectious aerosol samples were found from three of the 16 investigated individuals with SARS-CoV-2 RNA in exhaled air. Based on the emission rates of infectious viruses during singing, we modelled the time needed for a susceptible person to inhale one infectious dose if they were in the same room as someone who emits viruses. From the model results we conclude that the time scale for transmission of SARS-CoV-2 via aerosols can be as little as a few minutes in normal indoor environments.

### Infectivity of exhaled aerosol samples

**Figure 1:**
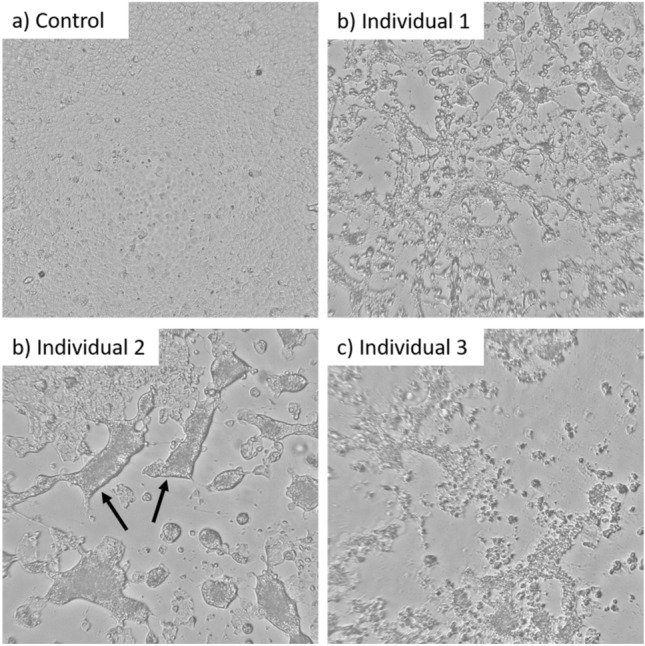
Cell cultures after 72 h incubation with (**a**) uninfected cells, and (**b**–**d**) aerosol samples (collected during singing) from SARS-CoV-2 infected individuals. Cytopathic effect is partly demonstrated as syncytia (examples indicated with arrows). Syncytia formation is known to be stronger in cell cultures infected by the alpha variant (individual 2) compared to the pre-alpha (individual 1 and 3)^13^.

Six aerosol samples from three individuals with covid-19 gave visual cytopathic effect (CPE) after 72 h using the qualitative culture assay (Fig. 1, Table 1). Aerosol samples from an additional 13 patients were culture-negative. From the three individuals with culture-positive aerosol samples, the aerosol samples collected during singing resulted in the highest infectivity: 2.5 × 10^4^, 7.9 × 10^3^, and 7.9 × 10^2^ TCID_50_/mL (Table 1). This corresponds to an emission rate in exhaled air of 127, 36 and 4 TCID_50_/s, respectively. The two culture-positive samples from talking (individual 1 and 2) both resulted in a TCID_50_/mL of 7.9 × 10^2^, which was the detection limit of the TCID_50_ assay, while the culture-positive sample from breathing (individual 3) was below the detection limit of the TCID_50_ assay.

**Table 1:** SARS-CoV-2 infectivity by the qualitative culture assay, corresponding RT-qPCR (Ct-values), and quantitative values of the TCID_50_ assay for different specimens.

Individuals (no 1–3)	Sample Ct	Culture result (+/−)	Supernatant Ct	TCID_50_/mL	Exhaled TCID_50_/s
Aerosol samples					
1: breathing	–^a^				
1: talking	37.5	+	12.3	7.9 × 10^2^	4
1: singing	35.3	+	13.2	2.5 × 10^4^	127
2: breathing	–^a^				
2: talking	35.7	+	12.5	7.9 × 10^2^	3
2: singing	33.7	+	18.7	7.9 × 10^3^	36
3: breathing	36.4	+	32.9	–	
3: talking	37.0	−	33.9	–	
3: singing	38.2	+	13.3	7.9 × 10^2^	4
NPH samples					
1	32.6^b^	+	16.5	–	
2	33.6^b^	+	13.0	7.9 × 10^2^	
3	35.2^b^	−	37.2	–	
Saliva samples					
1	39.3^c^	−	37.4	–^d^	
2	25.1^c^	+	17.4	–^d^	
3	18.6^c^	+	17.1	–^d^	

^a^No RNA detected and therefore not cultivated.

^b^Ct-value after filtration through 0.22 µm filter. Original sample Ct-values were: 30.4, 24.4 and 25.1

^c^Ct-value from Alsved et al.^12^.

^d^The saliva samples only had material for the 12-well assay, and no TCID_50_ assay could be performed.

The nasopharyngeal (NPH) samples of individual 1 and 2 showed CPE in the qualitative assay, but only that of individual 2 was quantifiable in the TCID_50_ assay, also at the detection limit 7.9 × 10^2^ TCID_50_/mL (Table 1). The saliva samples from individual 2 and 3 showed CPE in the qualitative assay, but quantification by TCID_50_ assay was not done due to sample shortage.

### Next generation sequencing of aerosol and NPH samples

Sequence analysis was performed on the NPH samples (original sample pre-cultivation) from individual 1 and 2 (sequencing of individual 3 NPH sample was not successful) as well as the post-cultivation supernatant of the singing samples from individuals 1, 2 and 3 (details in Table S1). Individual 1 and 3 were infected with pre-alpha variants and individual 2 with the alpha variant. This corresponded well to the morphology of the infected cells with stronger syncytia formation in cell cultures infected with the alpha variant (Fig. 1)^13^. Gene sequences from aerosol and NPH samples displayed an almost identical mutation pattern for individuals 1 and 2, respectively, as evidence of correct individual source of cultured viruses.

### Modelling time to inhale one infectious dose—the transient scenario

**Figure 2:**
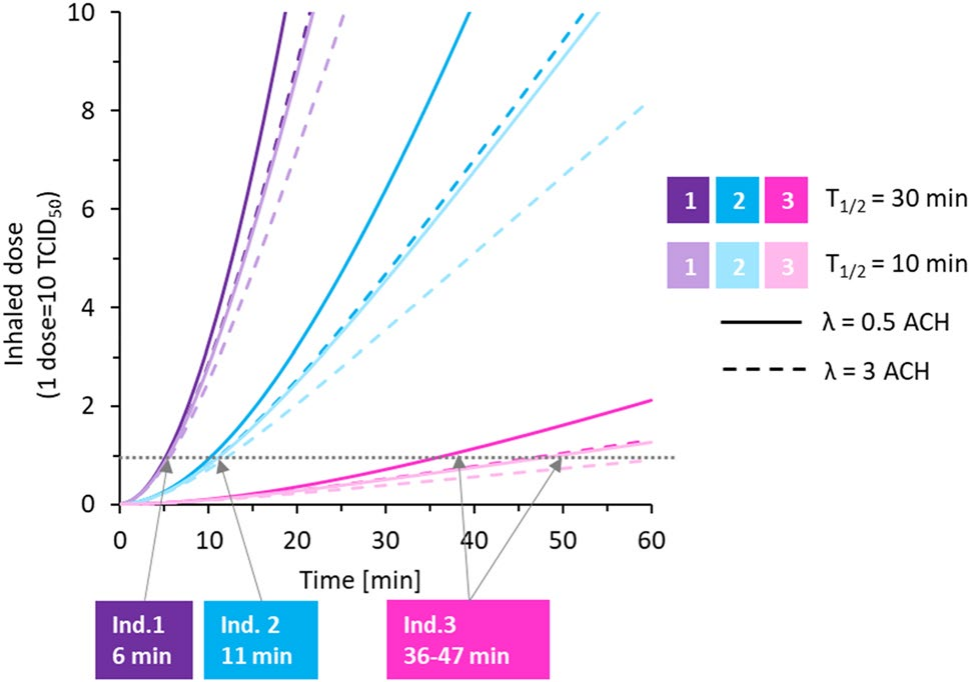
Inhaled infectious dose of SARS-CoV-2 in a susceptible adult as a function of time for the transient scenario where an infected individual enters a room and sings. The dotted horizontal line indicates one infectious dose, which corresponds to 10 TCID_50_,^10^ and the time to reach one dose is indicated for a half-life time, t_½_, of 30 min. Model input: room size = 4 × 4 × 3 m^3^, inhalation rate = 9 L/min.

We simulated a transient scenario where an infectious individual enters a previously virus-free room (at time = 0), and calculated the time required until another person in the room inhales one infectious dose (Fig. 2). We used the three emission rates for singing as the source in the indoor aerosol model and plotted the inhaled dose for the exposed person in the room as function of time for both normal ventilation, 0.5 ACH and enhanced ventilation, 3 ACH. Regardless of the room ventilation, one infectious dose would be inhaled within 6 or 11 min when individual 1 or 2, respectively, enters the room and sings. For individual 3, it would take 37 or 47 min with the normal or enhanced ventilation, respectively. The simulation was made for virus half-life times of both 10 and 30 min, but the difference in decay rates had limited impact.

### Modelling time to inhale one infectious dose—the steady-state scenario

Using the steady-state scenario where the infectious person has been staying (singing) in the room for a long time (1–3 h) before a susceptible person enters, the time needed to inhale one infectious dose is shorter than for the transient scenario as the concentration of viruses in the room air has reached steady-state from beginning of exposure. In the normally ventilated room scenario one dose would be inhaled in 1, 2 or 17 min when visiting individual 1, 2 and 3, respectively (Table 2, half-life time 30 min).

**Table 2:** Time to inhale one infectious dose when entering a room where one of the three infected individuals in this study has been staying (singing) long enough to reach steady-state concentration (~ 1 h in enhanced ventilation and ~ 3 h in the normal ventilation) of viruses in the air. Model input: room size = 4 × 4 × 3 m^3^, inhalation rate = 9 L/min.

Room conditions	Virus half-life time (min)	Steady-state concentration (TCID_50_/L room air) with source:			Time to inhale one dose^a^ (min) with source:		
		Ind. 1	Ind. 2	Ind. 3	Ind. 1	Ind. 2	Ind. 3
Normal (ventilation = 0.5 ACH)	10	1.9	0.5	0.06	1.2	4.3	38
	30	4.5	1.3	0.14	0.5	1.9	17
Well-ventilated (ventilation = 3 ACH)	10	1.3	0.4	0.04	1.8	6.4	57
	30	2.0	0.6	0.06	1.1	4.0	36

^a^1 dose = 10 TCID_50_.

**Figure 3:**
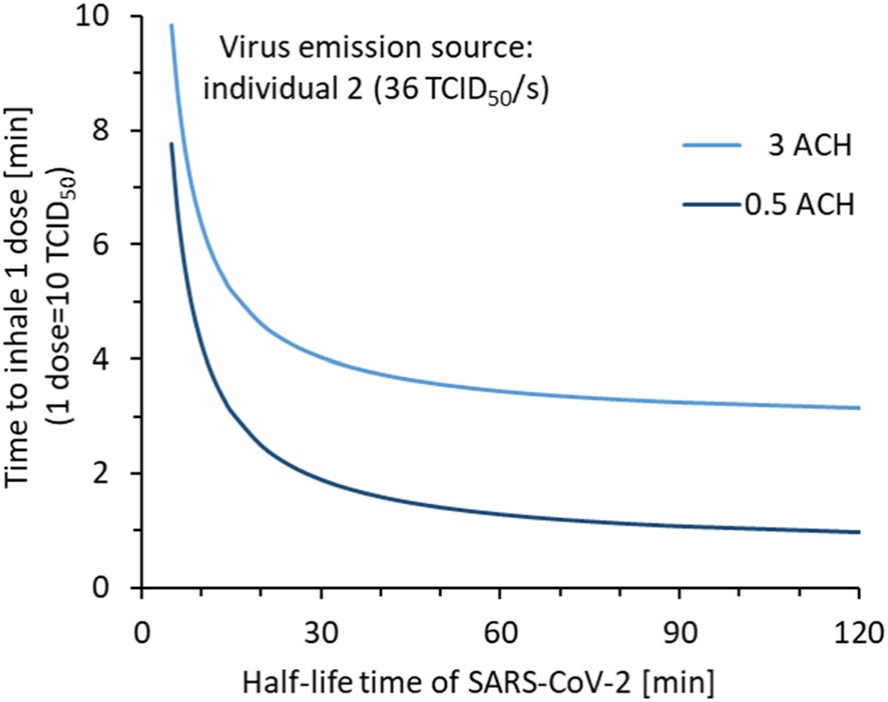
A sensitivity analysis of the impact of SARS-CoV-2 half-life time for virus viability in airborne state on the time it takes to inhale one infectious dose. Individual 2 singing was used as emission source.

The most uncertain parameter in the model is the half-life time of the airborne viruses. Therefore, we made a sensitivity analysis for the time needed to inhale one infectious dose with different virus viability half-life times (Fig. 3) in normal and enhanced ventilation. The time to inhale one infectious dose decreases rapidly as the half-life time increases from 5 to 30 min, indicating that if the half-life time is in this range, it has an essential impact on the inhaled dose. If instead, the half-life time is longer than 30 min, other processes such as physical removal by ventilation and deposition are more important. The infectious dose is another uncertain parameter, which inevitably changes the exposure time needed to acquire an infection. The time increases linearly with a higher infectious dose for the steady-state scenario, and close to linearly for the transient scenario (Fig. S1).

## Discussion

This study reports the first quantification of infectivity of SARS-CoV-2 in aerosols sampled directly from exhaled air. Aerosol samples were culturable from three of 16 individuals with detectable SARS-CoV-2 RNA in exhaled air. From these three individuals, five of six culturable aerosol samples were successfully quantified. The highest infectivity was found for samples collected close to symptom onset and during singing. Based on the culture results, we calculated the emission rate from the three individuals during singing and for two of them also for talking. The emission rates were thereafter implemented in an indoor air model for calculating the time needed for a susceptible person to inhale one infectious dose when being in the same room as the infectious person. This time can be as short as 6 min when a highly infectious individual enters the room or only 1 min if the infected person already has been in the room long enough to reach steady-state concentration of viruses in the air.

Previous studies have quantified infectivity of SARS-CoV-2 sampled from room air, but not directly from exhaled air^6–8^. Vass et al. found an infectivity of 132 and 192 plaque-forming units (PFU)/L air in two of five virus positive air samples in a residential setting. Kitagawa et al. detected RNA in the majority (12 of 18) of their air samples, and found five culturable samples with 0.58–10 TCID_50_/L of sampled air. Lednicky et al. reported viable virus in four of six air samples collected in a patient room, ranging from 6 to 74 TCID_50_/L of air. In comparison, the steady-state concentration in a room in our study would be 2.0, 0.6 and 0.06 TCID_50_/L air with the three individuals, respectively, at enhanced ventilation and a half-life time of 30 min (Table 2); thus, similar concentrations as those measured by Kitagawa et al.^8^ but lower values than those in Lednicky et al.^7^. However, they both had their aerosol samplers within one meter from the source where the concentration is likely enhanced, while our model assumes equal mixing in the room. For our simulated normal ventilation, which is more applicable for comparison with the measurement in Vass et al.^6^, the steady-state concentration would be 4.5, 1.3 and 0.14 TCID_50_/L air (Table 2). Although the room sizes, respiratory activities, experimental procedures and analysis differ between these previous measurements, it seems likely that the steady-state concentrations we simulated can be reached in a room with an infectious individual.

To verify the results, aerosol samples from singing and NPH samples were cultivated twice in the qualitative assay. The genome sequences showed high agreement between the supernatant of culture-positive aerosol samples and NPH samples, which suggests that air and nasal viruses originated from the same individuals. Moreover, the isolated virus types represented those circulating in the region at the time of sample collection (Feb–Mar 2021). CPE also matched with genotypes, where alpha variants are more prone to result in syncytia formation than pre-alpha variants^13^. The samples in the current study were transported for a few hours in outdoor temperature (5–10 °C) before storage at − 80 °C for 1 year, and were freeze-thawed at least once before cultivation. Thus, due to suboptimal sample handling there is a risk that we underestimated the infectivity of the culture-positive samples and the total number of culture-positive samples.

Although the RNA concentration of a sample is not directly related to its infectivity, RNA concentration has often been used as a proxy of infectivity or transmissibility in clinical settings. Remarkably, the culture-positive samples in our study all had relatively low levels of SARS-CoV-2 RNA (Ct-value range: 32–38) and the sample showing the highest infectivity was not the sample with the highest RNA concentration. In this study, the successful cultivation is partly attributed to the early phase of the infection^14,15^. The aerosol samples from individual 1 and 2, which had the highest TCID_50_ values, were collected on the day of symptom onset, which is when peak infectiousness is reached^16^, yet also when higher concentrations of SARS-CoV-2 RNA have been found in aerosol samples^8,11,12^. Transmission before and around symptom onset has been an important factor driving the covid-19 pandemic^17,18^, and a good predictor of infectivity is likely a combination of viral load and days from symptom onset.

Individual emission rates have strong influence over the calculated time to inhale one infectious dose (from 6 to 37 min in the transient scenario, Fig. 2). The enhanced ventilation (3 ACH instead of 0.5) is of less importance in the modelled indoor setting. Our indoor air model is based on an assumption of instant complete mixing of room air, i.e. that the concentration of airborne virus is similar at all places in the room. This assumption is a reasonable approximation for room sizes up to a few dozens of cubic meters. Still, on shorter time scales of seconds to minutes, the concentration is higher close to the source.

The time airborne viruses remain infectious is difficult to measure, and it is altered by the local environmental conditions such as temperature and humidity^19,20^. However, from the sensitivity analysis (Fig. 3) we can see that the half-life time of viruses is of less importance in well-ventilated rooms, as aerosol particles are physically removed prior to virus inactivation. Oswin et al. found that a substantial part of the infectivity is lost within the very first seconds in the air, presumably in the environmental transition from exhaled breath to room air conditions^20,21^. The aerosol samples cultivated in this study were collected after about 20 s from emission and after drying. Thus, we estimate that the large initial loss of infectivity had already happened before the point where we measure the TCID_50_.

We used the *ID*_*50*_ of 10 TCID_50_ that was identified in a human challenge study on SARS-CoV-2 for unvaccinated people^10^. The infectious dose in Killingley et al. was derived from cultivation in Vero E6 cells that did not express the transmembrane protease 2 (TMPRSS2). VeroE6/TMPRSS2 cells have been shown to have about ten-fold increased entry efficiency of SARS-CoV-2 caused by the TMPRSS2^22,23^. However, in the challenge study, SARS-CoV-2 was pipetted in the nose and hence, not inhaled via aerosols. For many viruses the infectious dose can be orders of magnitude lower via the aerosol route^24,25^. Our sensitivity analysis of the infectious dose (Fig. S1) shows that individual 1 and 2 are likely to transmit one infectious dose within 20–50 min also for an ID_50_ of 100 TCID_50_.

Our study includes the first model calculations based on measured source emission rates of exhaled viruses. However, previous studies have modelled well-documented superspreading events and estimated emission rates based on the number of people that became infected, by assuming aerosols as the only route of transmission^26,27^. Prentiss et al. analyzed six superspreading events with attack rates between 15 and 87% for which they assumed emission rates in the range 7.2 × 10^4^ to 1.1 × 10^6^ virions/h. This is in the same range as found here, if assuming 1 virion equals 1 TCID_50_ (4.6 * 10^5^, 1.3 × 10^5^ and 0.14 × 10^5^ TCID_50_/h for individuals 1–3, respectively). Their calculated *ID*_*50*_ (notated *N*_*0*_) was in the range of 300–2000. Reichert et al. measured particle emission rates from two index cases in choir superspreader events and calculated an *ID*_*50*_ of 12 virions^26^, which is similar to what was found in the human challenge study. They also predicted that one person would have inhaled one infectious dose within 8 min.

This study presents experimentally measured emission rates of infectious SARS-CoV-2 aerosols during breathing, talking and singing. When applying the measured emission rates in an indoor air particle transmission model, we found that an infectious dose is inhaled within a few minutes in a typical room with normal or enhanced ventilation. These findings demonstrate the potential of rapid aerosol transmission of SARS-CoV-2 in indoor environments.

## Methods

Exhaled aerosol samples from SARS-CoV-2-infected individuals were collected in Feb-Mar 2021 using a condensational growth tube collector (BioSpot-VIVAS, Aerosol Devices Inc. operating at 8 L/min) while the individuals were either breathing, talking or singing, respectively, for 10 min each as described previously^12^ (schematic setup shown in supplementary Fig. S2). SARS-CoV-2 RNA was detected in aerosol samples from 19 of the 38 included individuals^12^. The aerosol sample with the highest RNA concentration from each individual was considered for cell culture infectivity (two individuals were excluded due to sample transport issues and one due to sample shortage). When culture-positive aerosol samples were identified, all additional samples (irrespective of RNA levels) from the same individual were also cultivated. These samples included nasopharyngeal (NPH), saliva and additional aerosol samples (breathing, talking and singing). Cultivation of NPH and aerosol samples from singing was repeated once to verify the results.

The collection liquid in the BioSpot consisted of 1.5 mL sterile filtered phosphate buffered saline (PBS, Gibco) supplemented with 0.2 M sucrose (Sigma Aldrich) and 0.5 wt% bovine serum albumin fraction V (Sigma Aldrich) as used by Lednickyet al.^7^. NPH swabs were placed in 1.5 mL 140 mM buffered NaCl and filtered through a 0.22 µm filter before inoculation to avoid bacterial contamination in the cell culture.

### Quantification by RT-qPCR

Real-time reverse transcription polymerase chain reaction (RT-qPCR) was either performed as described previously^12^ or by a modified protocol (before and after cultivation to confirm replication). Total nucleic acid was extracted from 100 µL of samples using RNeasy Mini Kit (Qiagen, Hilden, Germany) according to the manufacturer’s instructions and eluted in 80 µL elution buffer. The RT-qPCR for the detection of SARS-CoV-2 RNA was performed on a QuantStudio 5 Real-Time PCR instrument (ThermoFisher Scientific, Massachusetts, USA) and all samples were tested in triplicates. Briefly, the reaction was performed in a 20 µL reaction mixture containing 5 µL of the extracted nucleic acids, 4× concentrated Ultraplex 1-step ThoughMix (Quantabio), 0.75 µM of each primer, 0.2 µM probe and 8.5 µL water. The assay is described in detail in^28^.

### Infectivity in cell cultures

Vero E6/TMPRSS2 cells (nibsc.org, product #100978) were grown in 12-well plates for 24 h to obtain an 80% confluent cell layer^22^. The cells were maintained in DMEM supplemented with 5% inactivated fetal calf serum (FCS), 100 units/mL of penicillin, 100 μg/mL of streptomycin and 500 μg/mL of geneticin. Each well was inoculated with 250 µL of collected aerosol sample and 250 µL DMEM with 2% FCS for 1 h at 37 °C and 5% CO_2_, and then 2 mL of DMEM with 2% FCS was added. After 72 h, 100 µL supernatant was removed for further analysis by RT-qPCR and next generation sequencing as previously described^29^. Sequences were analyzed using CLC Genomic Workbench 22 (Qiagen, Hilden, Germany) and mutations were analyzed with low frequency variant detection.

Culture-positive samples were evaluated with a standard median TCID_50_ assay in 96-well plates where 40 µL sample and 210 µL fresh media were added to each well. Ten-fold serial dilutions from 10^–1^ to 10^–8^ of the collected aerosol or NPH samples were inoculated in duplicates per dilution in 96-well plates. The plates were incubated at 37° C in a humidified 5% CO_2_ atmosphere and inspected daily for 4 days or until cytopathic effect (CPE) was observed. The TCID_50_ titers were determined when 50% of the cell cultures in wells showed a full CPE as compared to uninfected controls.

### Description of SARS-CoV-2 emitting individuals

Of the 16 SARS-CoV-2 emitting individuals included in this study, viruses could be cultured from three individuals (Table 3). Two of these individuals (number 1 and 2) were included on the day of symptom onset, showing mild symptoms. They were both quarantining at home due to covid-19 infected household contacts. Individual 1 reported no symptoms in the morning, mild symptoms during sample collection around noon and fever during the following night. Individual 2 was negative on rapid antigen test (Panbio COVID-19 antigen rapid test, Abbott) the day before sample collection, but was included in the study the following day when reporting mild sore throat and a repeated, now positive, antigen test. Individual 3 was exposed at work and when experiencing moderate symptoms, she tested positive by PCR, and was included 2 days from symptom onset. All three individuals fully recovered from the infection within 2 weeks. None of the individuals were previously vaccinated or had a known previous SARS-CoV-2 infection.

**Table 3:** Background data on the 16 individuals in our previous study^12^ whose aerosol samples were cultivated in this study, and the culture result (12-well CPE) of corresponding aerosol sample.

Individual no	Days from symptom onset	Culture result^a^ (+/−)	Age	Sex	Ct-value of aerosol sample	Ct-value of NPH sample
1	0	+	30	M	37	30
2	0	+	33	M	32	24
3	2	+	42	F	35	25
4	1	−	15	M	37	20
5	1	−	22	M	40	31
6	1	−	29	M	38	25
7	1	−	36	M	40	22
8	1	−	38	F	39	21
9	2	−	29	F	40	28
10	2	−	31	F	38	27
11	2	−	46	F	40	22
12	2	−	46	M	40	25
13	2	−	52	M	38	21
14	3	−	18	M	40	28
15	3	−	35	F	39	26
16	3	−	51	F	34	23

^a^Qualitative cultivation in 12-well plates.

### Model for calculation of inhaled dose

The indoor aerosol model used for estimating exposure and inhaled dose of SARS-CoV-2 has been described in detail previously^9^. It is based on mass-balance equations for the concentration of aerosol particles in the indoor environment and a respiratory tract particle deposition model to derive the inhaled dose of viruses. The model input parameters include indoor domain geometries, ventilation rate, dry deposition of particles on indoor surfaces, emission rate of viruses from the source (i.e. the infected person), aerosol particle size distribution, inhalation rate of the exposed individuals, gender specific respiratory tract deposition probability of the inhaled aerosol particles at different levels of physical activity and half-life time of the viruses (i.e. the time the viruses remain infectious in air).

The indoor scenario that was considered in this study for determining the time needed to inhale an infectious dose of SARS-CoV-2 was: room size of 4 × 4 × 3 m^3^; air exchange rate of either 0.5 ACH (air changes per hour, typical home environment) or 3 ACH (enhanced ventilation such as in some hospital areas and public buildings); aerosol particle size distribution according to Alsved et al.^11^ (with most viruses found in the range 1–4 µm, see Fig. S3); an average inhalation rate for men and women representing low activity (standing and sitting) of 9 L/min; emission rates of infectious viruses as found in the present study (model details in Supplement). Calculations were made both for a transient scenario, which corresponds to an infected individual entering a room with no previous viruses in the air where a susceptible person is exposed, and for a steady-state scenario, which corresponds to a susceptible person visiting a room where an infected individual has been for a time period of at least a few hours. The infectious dose of 10 TCID_50_ was taken from the human challenge study by Killingley, Mann^10^. One of the least known parameters in the model is the decay rate in infectivity of SARS-CoV-2 in air, and thus, the results are presented for half-life times of 10 and 30 min and a sensitivity analysis was made where virus half-life time was varied between 5 and 120 min.

### Ethics

All methods were carried out in accordance with relevant guidelines and regulations and conducted in accordance with the declaration of Helsinki. All participants received oral and written information and signed a written informed consent. This study protocol and experiments were approved by the Swedish Ethical Review Authority (case number 2020-07103).

### Supplementary Information

Supplementary Information.

## Data Availability

The datasets used and/or analyzed during the current study available from the corresponding author on reasonable request.

## References

[1] Meyerowitz EA, Richterman A, Gandhi RT, Sax PE (2021). Transmission of SARS-CoV-2: A review of viral, host, and environmental factors. Ann. Intern. Med..

[2] Duval D, Palmer JC, Tudge I (2022). Long distance airborne transmission of SARS-CoV-2: Rapid systematic review. BMJ.

[3] Coleman KK, Tay DJW, Tan KS (2022). Viral load of severe acute respiratory syndrome coronavirus 2 (SARS-CoV-2) in respiratory aerosols emitted by patients with coronavirus disease 2019 (COVID-19) while breathing, talking, and singing. Clin. Infect. Dis..

[4] Adenaiye OO, Lai J, Bueno de Mesquita PJ (2022). Infectious severe acute respiratory syndrome coronavirus 2 (SARS-CoV-2) in exhaled aerosols and efficacy of masks during early mild infection. Clin. Infect. Dis..

[5] Lai J, Coleman KK, Tai SHS (2022). Exhaled breath aerosol shedding of highly transmissible versus prior severe acute respiratory syndrome coronavirus 2 variants. Clin. Infect. Dis..

[6] Vass WB, Lednicky JA, Shankar SN, Fan ZH, Eiguren-Fernandez A, Wu CY (2022). Viable SARS-CoV-2 Delta variant detected in aerosols in a residential setting with a self-isolating college student with COVID-19. J. Aerosol. Sci..

[7] Lednicky JA, Lauzardo M, Fan ZH (2020). Viable SARS-CoV-2 in the air of a hospital room with COVID-19 patients. Int. J. Infect. Dis..

[8] Kitagawa H, Nomura T, Kaiki Y (2023). Viable SARS-CoV-2 detected in the air of hospital rooms of patients with COVID-19 with an early infection. Int. J. Infect. Dis..

[9] Hussein T, Londahl J, Thuresson S (2021). Indoor model simulation for COVID-19 transport and exposure. Int. J. Environ. Res. Public Health.

[10] Killingley B, Mann AJ, Kalinova M (2022). Safety, tolerability and viral kinetics during SARS-CoV-2 human challenge in young adults. Nat. Med..

[11] Alsved M, Nygren D, Thuresson S, Fraenkel C-J, Medstrand P, Löndahl J (2023). Size distribution of exhaled aerosol particles containing SARS-CoV-2 RNA. Infect. Dis..

[12] Alsved M, Nygren D, Thuresson S, Medstrand P, Fraenkel CJ, Löndahl J (2022). SARS-CoV-2 in exhaled aerosol particles from COVID-19 cases and its association to household transmission. Clin. Infect. Dis..

[13] Rajah MM, Hubert M, Bishop E (2021). SARS-CoV-2 alpha, beta, and delta variants display enhanced spike-mediated syncytia formation. EMBO J..

[14] Bullard J, Dust K, Funk D (2020). Predicting infectious severe acute respiratory syndrome coronavirus 2 from diagnostic samples. Clin. Infect. Dis..

[15] Singanayagam A, Patel M, Charlett A (2020). Duration of infectiousness and correlation with RT-PCR cycle threshold values in cases of COVID-19, England, January to May 2020. Eurosurveillance.

[16] Sun K, Wang W, Gao L (2021). Transmission heterogeneities, kinetics, and controllability of SARS-CoV-2. Science.

[17] Casey-Bryars M, Griffin J, McAloon C (2021). Presymptomatic transmission of SARS-CoV-2 infection: A secondary analysis using published data. BMJ Open.

[18] Johansson MA, Quandelacy TM, Kada S (2021). SARS-CoV-2 transmission from people without COVID-19 symptoms. JAMA Netw. Open.

[19] Tang JW (2009). The effect of environmental parameters on the survival of airborne infectious agents. J. R. Soc. Interface.

[20] Oswin HP, Haddrell AE, Otero-Fernandez M (2022). The dynamics of SARS-CoV-2 infectivity with changes in aerosol microenvironment. Proc. Natl. Acad. Sci..

[21] Löndahl J, Alsved M (2022). Abrupt decreases in infectivity of SARS-CoV-2 in aerosols. Proc. Natl. Acad. Sci..

[22] Matsuyama S, Nao N, Shirato K (2020). Enhanced isolation of SARS-CoV-2 by TMPRSS2-expressing cells. Proc. Natl. Acad. Sci..

[23] Bruce EA, Mills MG, Sampoleo R (2022). Predicting infectivity: Comparing four PCR-based assays to detect culturable SARS-CoV-2 in clinical samples. EMBO Mol. Med..

[24] Yezli S, Otter JA (2011). Minimum infective dose of the major human respiratory and enteric viruses transmitted through food and the environment. Food Environ. Virol..

[25] Weber TP, Stilianakis NI (2008). Inactivation of influenza A viruses in the environment and modes of transmission: A critical review. J. Infect..

[26] Reichert F, Stier O, Hartmann A (2022). Analysis of two choir outbreaks acting in concert to characterize long-range transmission risks through SARS-CoV-2, Berlin, Germany, 2020. PLoS ONE.

[27] Prentiss M, Chu A, Berggren KK (2022). Finding the infectious dose for COVID-19 by applying an airborne-transmission model to superspreader events. PLoS ONE.

[28] Wang H, Churqui MP, Tunovic T (2022). The amount of SARS-CoV-2 RNA in wastewater relates to the development of the pandemic and its burden on the health system. iScience.

[29] Beck-Friis T, Kärmander A, Nyström K (2022). Comparison of SARS-CoV-2 spike RNA sequences in feces and nasopharynx indicates intestinal replication. Gut Pathog..

