# Supplementary material for: Infectivity of exhaled SARS-CoV-2 aerosols is sufficient to transmit covid-19 within minutes
Source: Sci Rep. 2023 Dec 1;13:21245. doi: 10.1038/s41598-023-47829-8 (PMC10692216; doi:10.1038/s41598-023-47829-8)
Supplement: Supplementary file 1 — Supplementary Information. [file 41598_2023_47829_MOESM1_ESM.pdf]

Supplementary Information to:

Infectivity of exhaled SARS-CoV-2 aerosols is sufficient to transmit covid-19 within minutes

Malin Alsved, Kristina Nyström, Sara Thuresson, David Nygren, Marianela Patzi-Churqui, Tareq Hussein, Carl-Johan Fraenkel, Patrik Medstrand, Jakob Löndahl

Next generation sequencing analysis

Table S1. Next generation sequencing analysis of aerosol sample supernatants post-cultivation and (original pre-cultivation) NPH samples showing mutations in the spike region.

**Sample**	**Source**	**Mutations**
Individual 1	Aerosol	99ns, A222V, D614G
Individual 1	NPH	99ns, A222V, D614G
Individual 2	Aerosol	69-70del, 144del, W152R, N501Y, A570D, D614G, P681H, T716I, T739I, S982A, D1118H
Individual 2	NPH	69-70del, 144del, W152R, N501Y, A570D, D614G, P681H, T716I, T739I (19%), S982A, D1118H
Individual 3	Aerosol	20ns, A222V, 375ns, D614G, A1078S
Individual 3	NPH	(unsuccessful amplification)

ns – nonsynonymous mutation, del – amino acid deletion

Sensitivity analysis of the infectious dose

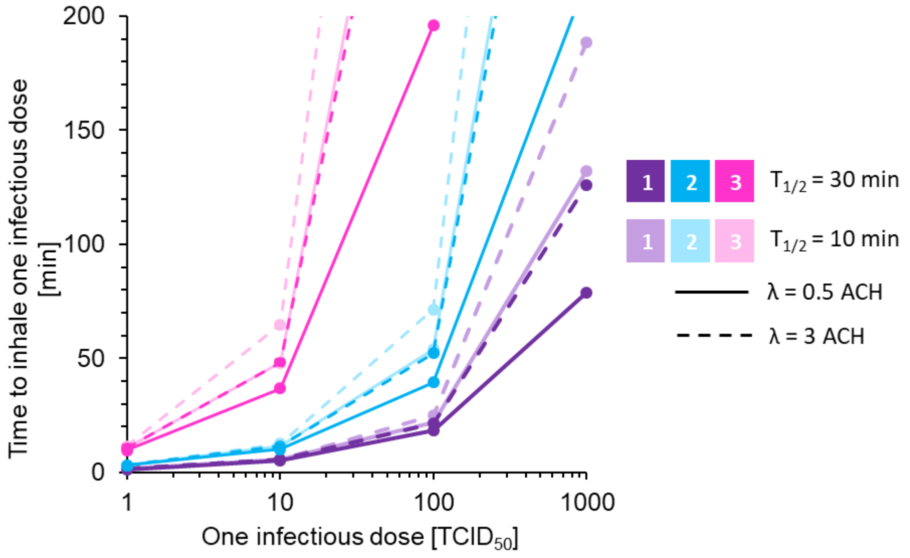

Figure S1. Sensitivity analysis of the infectious dose on the modelled exposure time needed to acquire one infectious dose in the transient scenario. Data is presented for having either individual 1 (purple), 2 (blue) or 3 (pink) as the emitting source, for two half-life times 10 and 30 min, and for the two ventilation air change rates (ach) of 0.5 and 3. For individual 1 and 2, the time to inhale one infectious dose is within one hour up to an infectious dose of 100 $TCID_{50}$.

Aerosol collection setup

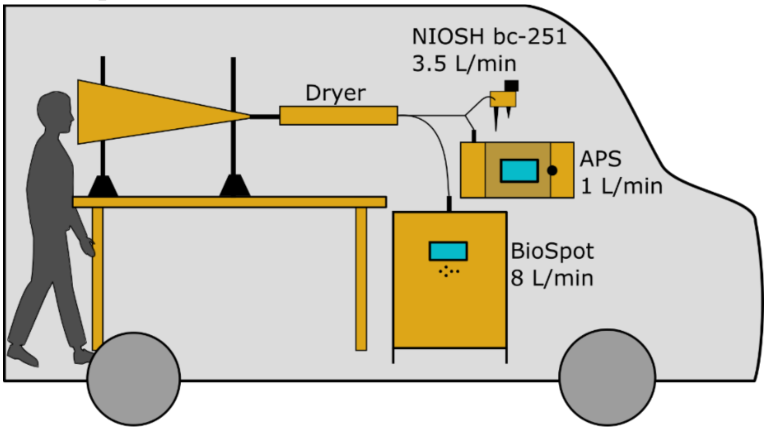

Figure S2. Experimental setup for collection of aerosol samples from the exhaled breath of individuals with covid-19. The back of the truck was open to outdoor air during sampling. The samples cultured in this study were collected with the BioSpot into 1.5 mL sterile filtered phosphate buffered saline supplemented with 0.2 M sucrose and 0.5 wt% bovine serum albumin fraction V. APS: aerodynamic particle sizer.

Model input data

Indoor Air Model parameters:
Room Volume, V = 4x4x3 m^3
Outdoor concentration, O = 0 $TCID_{50}/cm^3$
Initial Indoor concentration, I_0 = 0 $TCID_{50}/cm^3$
Penetration factor, P = 0
Ventilation rate, l = 0.5 or 3 h^{-1}
Friction velocity, u* = 0.1 m/s
Emission rate: 127, 36 or 4 $TCID_{50}$/s
Half-life time of the virus: 10 or 30 min
Inhalation rate (average for males and females, standing and sitting): 9 L/min
Virus distribution in aerosol particles of various sizes: see Figure S3

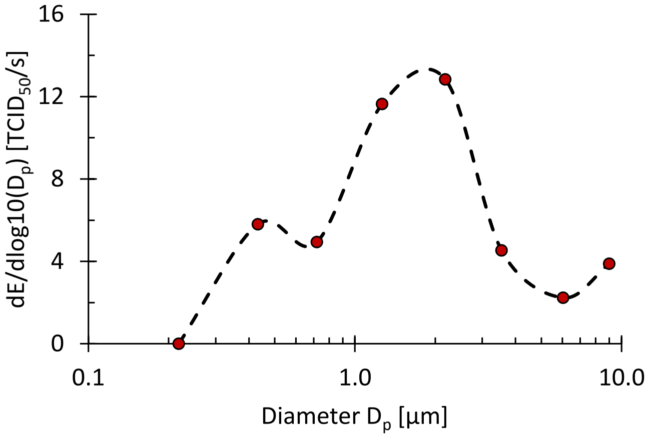

Figure S3. Distribution of virus in exhaled aerosol particles, derived from Alsved et al. (2023) including all three respiratory exercises (breathing, talking and singing).

The virus distribution in exhaled aerosol particles match the shape of the mass- or volume distribution of exhaled aerosols. In the number distribution, the first mode at ~0.4 µm is the highest and the second mode at 1-2 µm is lower.
